# Biochemical properties of novel Carbon nanodot-stabilized silver nanoparticles enriched calcium hydroxide endodontic sealer

**DOI:** 10.1371/journal.pone.0303808

**Published:** 2024-07-03

**Authors:** Tayyaba Waqar Ali, Hashmat Gul, Muhammad Amber Fareed, Sobia Tabassum, Sana Rubab Mir, Aqsa Afzaal, Nawshad Muhammad, Muhammad Kaleem

**Affiliations:** 1 Department of Dental Materials, Army Medical College, National University of Medical Sciences (NUMS), Rawalpindi, Pakistan; 2 Department of Clinical Sciences, College of Dentistry, Ajman University, Ajman, UAE; 3 Center of Medical and Bio-allied Health Sciences Research, Ajman University, Ajman, UAE; 4 Interdisciplinary Research Centre in Biomedical Materials (IRCBM), COMSATS University Islamabad, Lahore Campus, Lahore, Pakistan; 5 Department of Chemistry, COMSATS University Islamabad, Lahore Campus, Lahore, Pakistan; 6 Department of Dental Materials, Institute of Basic Medical Sciences, Khyber Medical University, Peshawar, Khyber Pakhtunkhwa, Pakistan; University of Sulaimani College of Dentistry, IRAQ

## Abstract

Calcium Hydroxide-based endodontic sealer loaded with antimicrobial agents have been commonly employed in conventional root canal treatment. These sealers are not effective against *E*. *faecalis* due to the persistent nature of this bacterium and its ability to evade the antibacterial action of calcium hydroxide. Therefore, endodontic sealer containing Carbon nanodots stabilized silver nanoparticles (CD-AgNPs) was proposed to combat *E*. *faecalis*. The therapeutic effect of CD-AgNPs was investigated and a new cytocompatible Calcium Hydroxide-based endodontic sealer enriched with CD-AgNPs was synthesized that exhibited a steady release of Ag+ ions and lower water solubility at 24 hours, and enhanced antibacterial potential against *E*. *faecalis*. CD-AgNPs was synthesized and characterized morphologically and compositionally by Scanning Electron Microscopy, Fourier Transform Infrared Spectroscopy (FTIR), and UV-Vis Spectroscopy, followed by optimization via minimum inhibitory concentration (MIC) determination against *E*. *faecalis* by broth microdilution technique and Cytotoxicity analysis against *NIH3T3* cell lines via Alamar Blue assay. Calcium hydroxide in distilled water was taken as control (C), Calcium hydroxide with to CD-AgNPs (5mg/ml and 10mg/ml) yielded novel endodontic sealers (E1 and E2). Morphological and chemical analysis of the novel sealers were done by SEM and FTIR; followed by *in vitro* assessment for antibacterial potential against *E*. *faecalis* via agar disc diffusion method, release of Ag+ ions for 21 days by Atomic Absorption Spectrophotometry and water solubility by weight change for 21 days. CD-AgNPs were 15–20 nm spherical-shaped particles in uniformly distributed clusters and revealed presence of constituent elements in nano-assembly. FTIR spectra revealed absorption peaks that correspond to various functional groups. UV-Vis absorption spectra showed prominent peaks that correspond to Carbon nanodots and Silver nanoparticles. CD-AgNPs exhibited MIC value of 5mg/ml and cytocompatibility of 84.47% with *NIH3T3* cell lines. Novel endodontic sealer cut-discs revealed irregular, hexagonal particles (100–120 nm) with aggregation and rough structure with the presence of constituent elements. FTIR spectra of novel endodontic sealers revealed absorption peaks that correspond to various functional groups. Novel endodontic sealers exhibited enhanced antibacterial potential where E-2 showed greatest inhibition zone against *E*. *faecalis* (6.3±2 mm), a steady but highest release of Ag+ ions was exhibited by E-1 (0.043±0.0001 mg/mL) and showed water solubility of <3% at 24 hours where E-2 showed minimal weight loss at all time intervals. Novel endodontic sealers were cytocompatible and showed enhanced antibacterial potential against *E*. *faecalis*, however, E2 outperformed in this study in all aspects.

## Introduction

Endodontics deals with the dental pulp and the tissues that surround the roots of teeth. Endodontic infections are treated with various methods, such as vital pulp therapy, regular and surgical endodontic treatment [1]. When the dental pulp is vital, it can be treated using vital pulp therapy but if it loses its vitality, root canal treatment becomes necessary [2]. Endodontic sealers along with core obturation materials seal the prepared root canal space [3]. Several endodontic sealers are available including salicylate-based, zinc oxide-eugenol, epoxy resin-based, glass ionomer-based, silicone and methacrylate resin-based endodontic sealers [3]. Calcium hydroxide sealer has widespread use in endodontics due to its well-established antibacterial and biological properties. American Association of Endodontists defines Calcium Hydroxide as a strongly basic, odorless, white powder often used in nonsurgical endodontic procedures as an intracanal medicament [4].

Root canal infection has a polymicrobial nature with *Enterococcus faecalis* being one of the primary microorganisms responsible because of the formation of several proteolytic enzymes and destructive aggregation constituents [5]. *E*. *faecalis* show resistance to calcium hydroxide alone and thus, is the cause of secondary and persistent endodontic infections [6]. To combat *E*. *faecalis*, various antimicrobial agents are added to calcium hydroxide sealer including antibiotics such as Metronidazole, Minocycline, and Vancomycin [7]. Due to the problem of development of antibiotic resistance in microbes, it has become mandatory to develop novel antimicrobial products to treat diseases caused by these drug-resistant microorganisms [8]. Thus, there is a need to incorporate novel antimicrobial agents such as nanoparticles into endodontic sealers [9]. Silver nanoparticles show significant antibacterial properties against *E*. *faecalis* [5]. Upon combination of Silver nanoparticles with calcium hydroxide, the antimicrobial activity was enhances as compared to calcium hydroxide alone [10]. There are concerns regarding the harmfulness of Silver nanoparticles because of creation of Reactive Oxygen Species (ROS) resulting in membrane degradation [8]. Carbon nanodots, a unique set of nanomaterials that are prepared from different carbon-based resources [11]. Carbon nanodots are bioactive and they are added to Silver nanoparticles to scavenge the ROS and thus, stabilize them [12].

In this study Carbon nanodots-stabilized Silver nanoparticles (CD-AgNPs) are synthesized and characterized of followed by their minimum inhibitory concentration (MIC) determination against *E*. *faecali*s and cytocompatibility with NIH3T3 cell lines. Novel calcium hydroxide based endodontic sealers containing CD-AgNPs were also synthesize and characterized by *in-vitro* testing to analyze the antibacterial properties and degradation profile via water solubility and release kinetics.

## Materials and methods

Analytical grade materials utilized in this study included calcium hydroxide (VWR International Ltd, Avantor), Brain Heart Infusion Broth (Thermo Scientific, Catalog#BC0100M), Mueller-Hinton Agar (Sigma Aldrich, Lot#3226573), Nitric acid (Sigma Aldrich, Lot#K28240743 037) whereas Bacterium *E*. *faecalis* was obtained from Musaji Adam and Sons (ATCC 29212).

### Synthesis and optimization of CD-AgNPs

Synthesis of CD-AgNPs was done in two steps. First, the synthesis of Carbon nanodots was done by carbonization of banana peels in an oven at 80°C for 12 hr which was then ground into fine powder and added to deionized water. The resultant solution was heated in the microwave and filtered through a filter paper. Finally, centrifugation was done at 5000 rpm for 10 hr followed by freeze-drying [13]. In second step, equal amounts of an aqueous solution of produced Carbon nanodots (0.06 mg/ml) and silver nitrate (AgNO_3_) solution (0.01M) were stirred together at room temperature. At varying concentrations of AgNO_3_ solution and different levels of pH, the synthesis of the prepared CD-AgNPs was optimized [13] via MIC determination against *E*. *faecalis* by broth microdilution technique and Cytotoxicity analysis against NIH3T3 cell lines via Alamar Blue assay.

### Minimum inhibitory concentration (MIC) determination

MIC of CD-stabilized AgNPs against *E*. *faecalis* was determined by broth microdilution susceptibility test using Brain Heart Infusion broth by following the methodology of CLSI M07-A10 [14, 15]. Serial two-fold dilutions of CD-stabilized AgNPs solution were prepared. The concentration of the stock solution was 5mg/ml. For preparation of *E*. *faecalis* Inoculum, nutrient-rich agar plates were taken and streaked with bacterial isolates of *E*. *faecalis* to obtain bacterial colonies. The plates were then incubated for 18–24 hours at 37 ˚C. Growth method was used for the preparation of the inoculum. From the agar plate culture, four to five well-isolated colonies of similar morphology were taken. Sterile cotton swabs were used to touch each colony. The growth was then shifted to a test tube holding 5ml BHI broth and suspension’s turbidity was adjusted to that of a 0.5 McFarland standard. This was done by the addition of more bacterial material if the turbidity was too low or by the addition of sterile broth if the turbidity was too high. The bacterial inoculum was then used within 30 minutes of turbidity adjustment to avoid change in the cell number. Suspension comprising around 1 to 2×10^8^ colony-forming units (CFU) per ml was obtained [16].

### Cytotoxicity testing

Cytotoxicity testing of the CD-AgNPs was performed by employing the Alamar blue assay after seeking ethical approval from Institutional Review Board, National University of Medical Sciences. NIH3T3 cell lines were first cultured twice in 10% fetal bovine serum (FBS) enriched Dulbecco’s modified eagle’s medium (DMEM) at 37°C and 5% CO_2_. Medium holding NIH3T3 cells was then introduced to 96-well plates. The cells were allowed to fully adhere to the plate. Then the original cell culture medium was swapped by the extracted substance. Cell incubation was done at 37°C with 5% CO_2_ for 1, 3 and 7 days [17].

### Grouping of novel endodontic sealers

Based on the findings of MIC and cytotoxicity testing of CD-stabilized AgNPs, only two concentrations with least cytotoxicity but acceptable anti-bacterial potential against *E*. *faecalis* were selected for this study. Grouping of novel endodontic sealers were then done based on their liquid components as shown in Table 1. Plain Calcium Hydroxide powder with the respective liquid components were used to prepare the required disc samples (7 mm diameter and 3mm height). The details of the groups employed in this study are given in **Table 1.**

**Table 1:** Composition of experimental endodontic sealers and control group.

Groups	Groups Names	Groups Composition	
		Powder	Liquid
**Control group**	**C**	Pure (100%) Calcium Hydroxide	Distilled Water
**Experimental group 1**	**E1**	Pure (100%) Calcium Hydroxide	CD-AgNPs in concentration of 5mg/ml
**Experimental group 2**	**E2**	Pure (100%) Calcium Hydroxide	CD-AgNPs in concentration of 10mg/ml

### Characterization of CD-AgNPs stock solution and the experimental endodontic sealers

#### Scanning electron microscopy

Nova NanoSEM 450 field-emission scanning electron microscope (FE-SEM) was employed at 1kV voltage, 1.4 nm resolution in high vacuum mode, to evaluate morphology and distribution of the CD-AgNPs in its stock solution (5 mg/ml) as well as within the experimental endodontic sealers i.e., E-1 and E-2 sample discs.

Elemental analysis of the prepared CD-AgNPs stock solution (5 mg/ml) and the cut sample discs of the experimental endodontic sealer groups i.e., E1 and E2, were carried out via Energy Dispersive X-Ray Spectroscopy (EDS) linked with SEM at 1kV and 1.4nm resolution in high vacuum mode. Everhart-Thornley Detector was used.

#### Fourier transform infrared (FTIR) spectroscopy

Thermo Scientific Nicolet 6700 FTIR spectrometer at 600–4000cm^−1^ range in ATR mode was used to obtain FTIR spectra for Carbon nanodots, CD-AgNPs and experimental endodontic sealers i.e., E1 and E2.

#### Ultraviolet–visible (UV–Vis) spectrophotometry

Ultraviolet–visible (UV–Vis) spectrophotometry of CD-AgNPs was done by UV-Visible Spectrophotometer (Perkin Elmer Lambda 25). The spectral range was from 200 to 400 nm and the path length was 10 mm.

### *In-vitro* testing of experimental endodontic sealers

#### Sample preparation

For sample preparation of control group, 400 μg of pure calcium hydroxide powder (VWR Chemicals) was mixed with 400 μl distilled water on a glass slab with a mixing spatula to produce a fine paste which was then scooped up and poured into a circular Teflon mold (7 mm diameter and 3mm height) placed on a Mylar sheet. Sample discs of experimental groups E1 and E2 were prepared in a similar fashion except that instead of distilled water, 400μl of CD-AgNPs solutions in the concentration of 5 mg/ml and 10 mg/ml respectively were used with calcium hydroxide powder.

#### Antibacterial analysis

Antibacterial activity of the endodontic sealers was assessed by Agar well diffusion [18]. Six Petri dishes containing 25 ml Brain heart infusion agar were taken. 100 μl bacterial medium was lawned onto three petri dishes homogenously and 5 wells (3mm diameter and 3mm depth) were prepared in each Petri dish. In these agar plates, 4 wells were brimmed with 21 μl of the different compositions of the sealers and the 5^th^ well was brimmed with plain calcium hydroxide which served as control. 2 agar plates were left empty (positive control) and 1 plate was loaded with sealers, but with no bacterial medium (negative control). This was done to determine and verify the sealer’s cleanliness. Agar petri dishes were then incubated at 35°C, for 16–20 hours [19] followed by measurement of inhibition zones in millimeters using a vernier caliper. The zones of inhibition (ZOI) were measured by subtracting the well diameter from half diameter of the ZOI.

#### Evaluation of silver release kinetics

Release characteristics of silver present in endodontic sealer samples discs were analyzed for 21 days by a specific recommended technique [20] using Atomic Absorption Spectrophotometer (Hitachi Z-5000) in flame mode at a wavelength of 413 nm. In 10 ml 68% concentrated nitric acid solution (Merc Ltd), disc samples (7 mm diameter and 3 mm height) of groups E1 and E2 were then stored. At planned time intervals i.e., day 1, day 7, day 14 and day 28, 1 ml storage solution was pipetted out and replaced by 1 ml fresh storage solution. The 1 ml pipetted out storage solution was then stirred in 100 μl nitric acid followed by addition of distilled water to achieve 5 ml final volume. Absorbance unit (Au) was used to measure absorbance.

#### Evaluation of water solubility

Evaluation of water solubility was done by immersing samples of control and experimental groups in distilled water. The samples were periodically analyzed at the following time intervals i.e., day 1, day 7, day 14, and day 21. At each time interval, the samples were picked out of distilled water and blotted with a filter paper to dry them completely followed by weighing them on an electronic balance. Following equation was utilized to calculate water solubility:

W(%)=((M0−M)÷(M0))×100

where, W = Water Solubility; M0 = Initial mass of sample immediately after removal from Teflon moulds; and M = Final mass of the sample after storage at 37°C in dry circumstances until equilibrium is reached [21].

### Statistical analysis

Collected data was statistically analyzed via statistical package for social sciences (SPSS) software version 22. Means and standard deviations of numerical variables were presented as bar graphs. Two-way ANOVA and post hoc Tukey test were done to evaluate the statistically significant difference within and between the groups for the variables (release kinetics of Silver nanoparticles and water solubility) at planned time intervals. Statistical significance difference in antibacterial activity within and between groups was evaluated using one-way ANOVA and post hoc Tukey tests. *p-value* of ≤ 0.05 was taken as statistically significant.

## Results

### MIC determination of CD-stabilized AgNPs

The MIC of CD-stabilized AgNPs was found to be 5 mg/ml, the microtiter plate showed positive control in column 1 and negative control in column 12 whereas columns 3–11 showed serial twofold dilutions of the CD-stabilized AgNPs stock solution.

### Cytotoxicity index of CD-stabilized AgNPs

A statistically significant difference in cytotoxicity was observed within and between all groups of the prepared solutions for this study *(p value = 0*.*000)*. Maximum number of viable cells i.e., NIH3T3 cells, were shown by control group (99.99±0.01%) whereas the number of viable cells reported for both concentrations of CD-stabilized AgNPs solutions i.e., E1 (5mg/mL CD-stabilized AgNPs) and E2 (10mg/mL CD-stabilized AgNPs) were 84.47±0.82% and 82.84±0.43% respectively, thus, indicating that the synthesized concentrations of CD-stabilized AgNPs solutions were biocompatible in relation to the control group.

### Scanning electron microscopy

**Fig 1:**
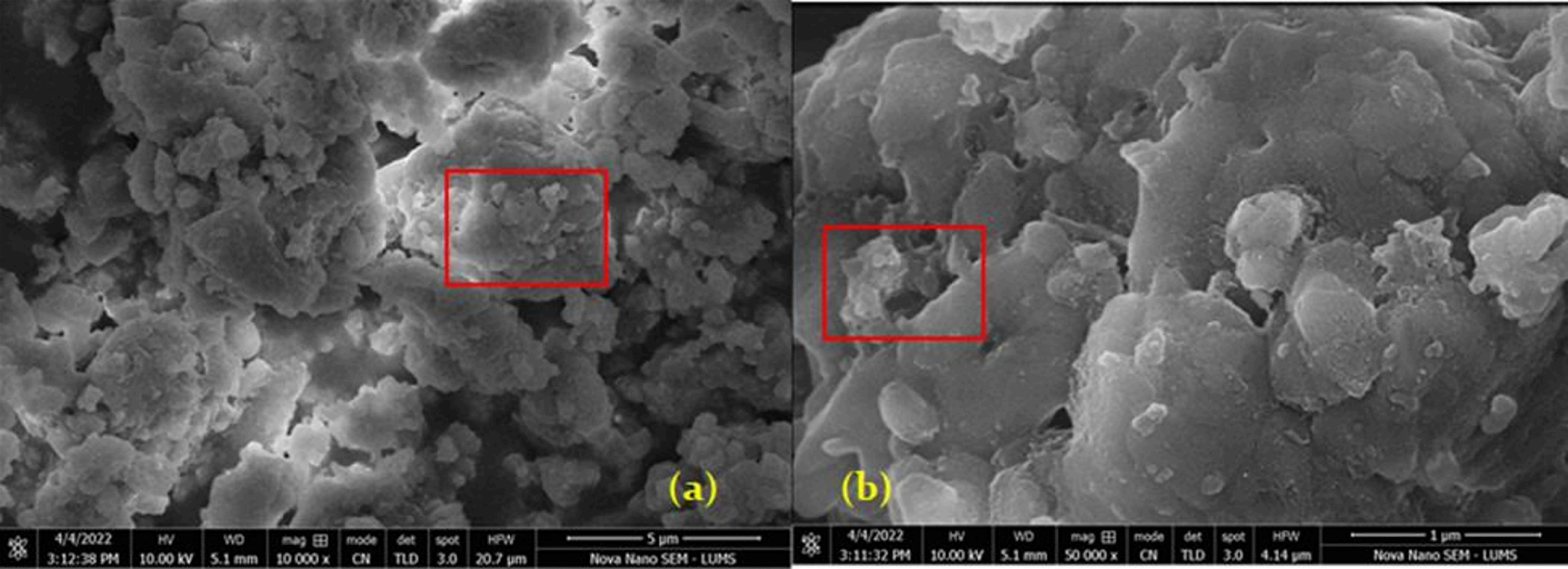
SEM of CD-AgNPs (a) at a magnification of 10,000X- Highlighted area shows irregular clusters of Silver nanoparticles (b) at a magnification of 50,000X- windows indicate aggregation of nanoparticles.

The stock solution of CD-AgNPs (5mg/ml) revealed clusters of uniformly distributed nanoparticles. Nanoparticles were found to have a spherical shape **(Fig 1).** As per ImageJ, the size of nanoparticles was 15–20 nm. EDS analysis of CD-AgNPs solutions (Table 2) verified the presence of Silver, Carbon and Oxygen in this material.

**Table 2:** EDS analysis of CD-AgNPs showing the average weight percentages of all the constituent elements.

Element	Atomic No. (%)	Mass (%)	Mass Norm (%)	Atom (%)	Abs. Error (%)	Rel. Error (%)
**Oxygen**	8	8.63	66.26	76.90	1.23	14.23
**Carbon**	6	2.02	15.49	12.51	0.17	8.30
**Silver**	47	0.74	5.68	0.98	0.06	7.68

**Fig 2:**
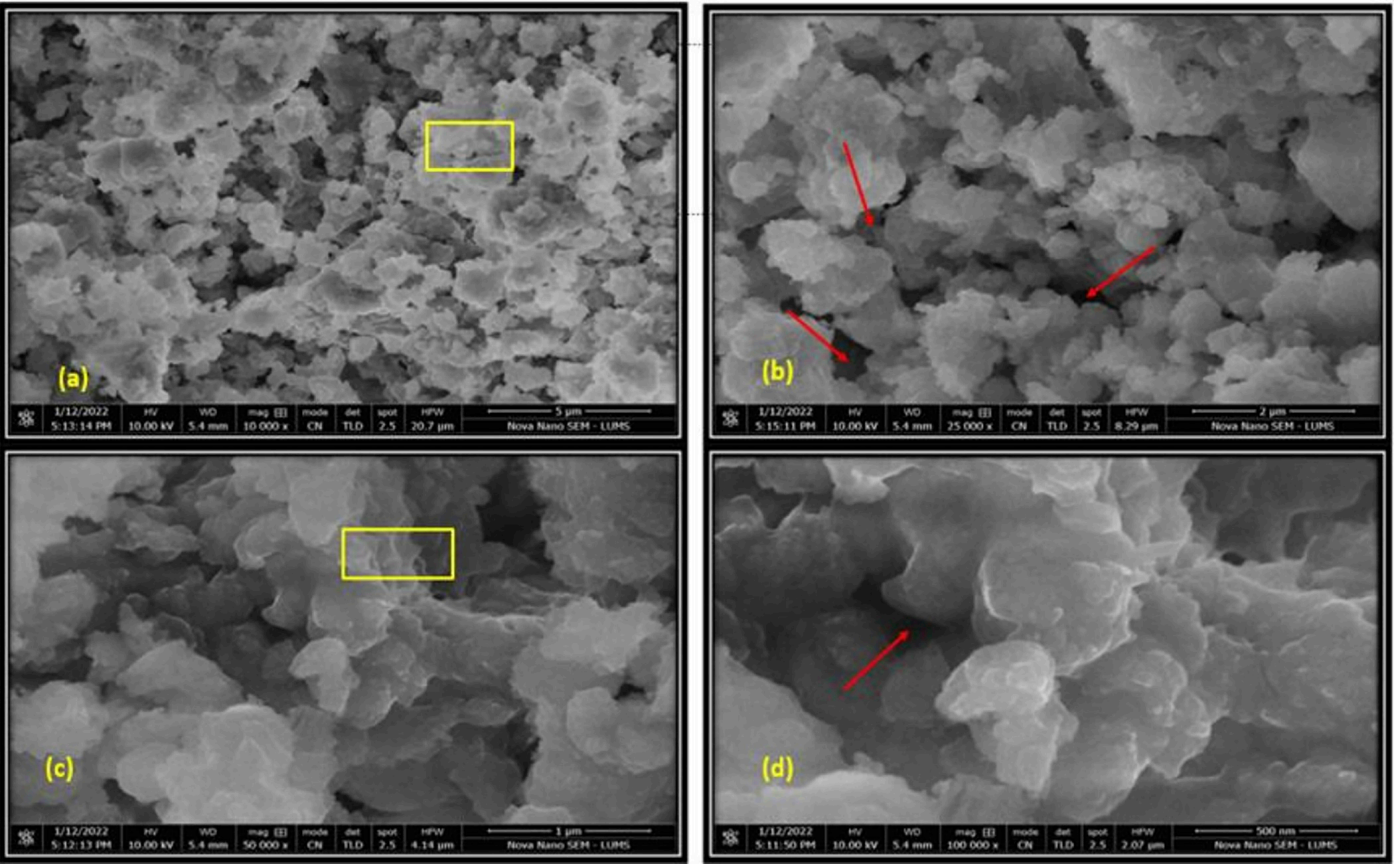
SEM of endodontic sealer sample disc, E1; windows showing aggregation whereas arrows indicate channels and pores.

**Fig 3:**
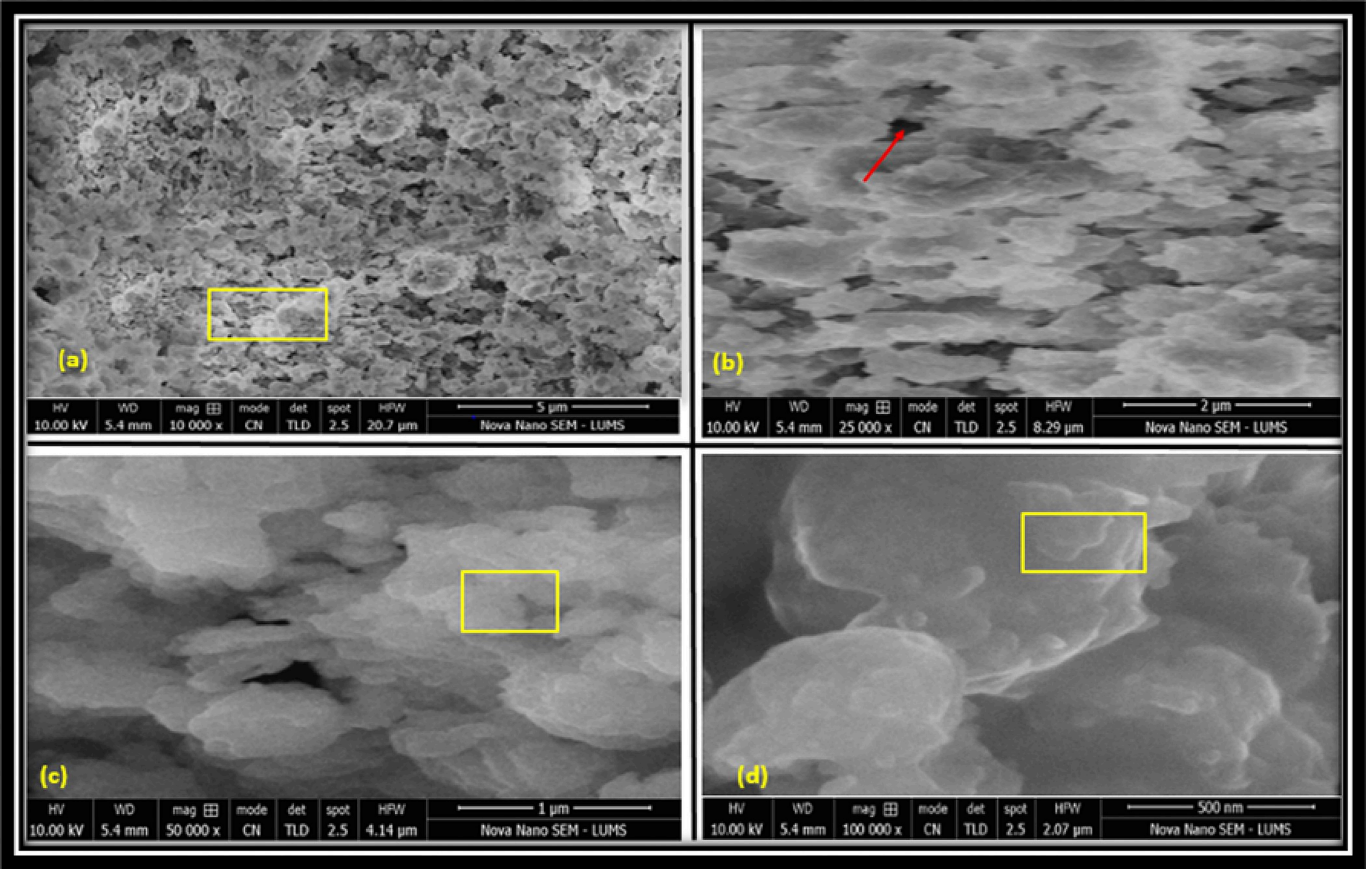
SEM of endodontic sealer sample disc, E2; windows showing aggregation whereas arrows indicate channels and pores.

SEM images of E-1 and E-2 endodontic sealer disc samples, at different resolutions (Figs 2 and 3), revealed uneven / rough surfaces along with the presence of channels and pores. E-2’s surface was rougher than E-1. Moreover, E-1 revealed more porosities as compared to E-2. CD-AgNPs were found to be hexagonal, were 100–120 nm in size, and showed aggregation.

EDS analysis of sample discs of the groups of experimental endodontic sealers E-1 and E-2 confirm the presence of Carbon, Calcium, Silver, and Oxygen in these materials (**Table 3**).

**Table 3:** EDS analysis of cut-disc samples of experimental endodontic sealers i.e., E-1 and E-2 showing the average weight percentages of all the constituent elements.

S.No.	Groups	Elements			
		Carbon (C)	Oxygen (O)	Silver (Ag)	Calcium (Ca)
1.	E-1	10.93%	51.65%	1.97%	35.45%
2.	E-2	12.93%	49.65%	1.98%	35.44%

**Fig 4:**
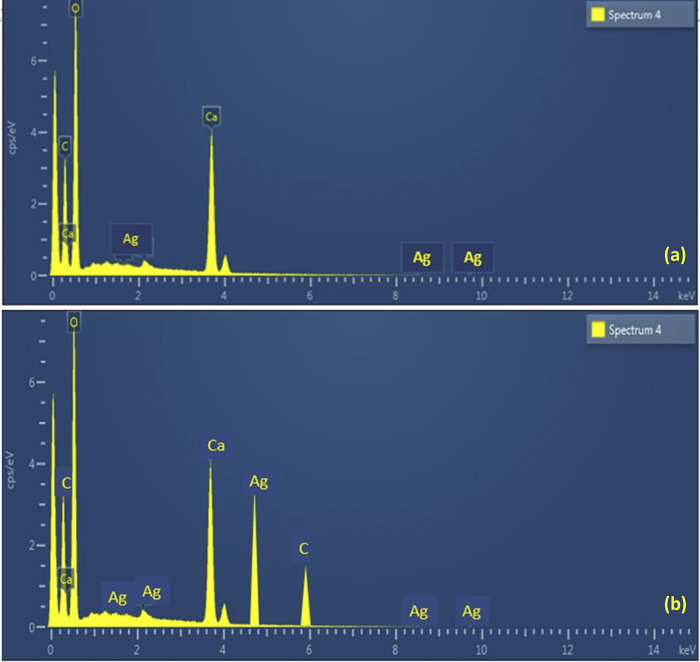
EDS analysis of sample discs, (a) group E-1, (b) group E2.

Fig 4(A) and 4(B) depicts the EDS analysis of sample discs of the groups of experimental endodontic sealers E-1 and E-2 respectively. Both figures confirm the presence of Carbon, Silver and Oxygen in the synthesized endodontic sealers.

### UV-Vis spectroscopy of CDs and CD-stabilized AgNPs

**Fig 5:**
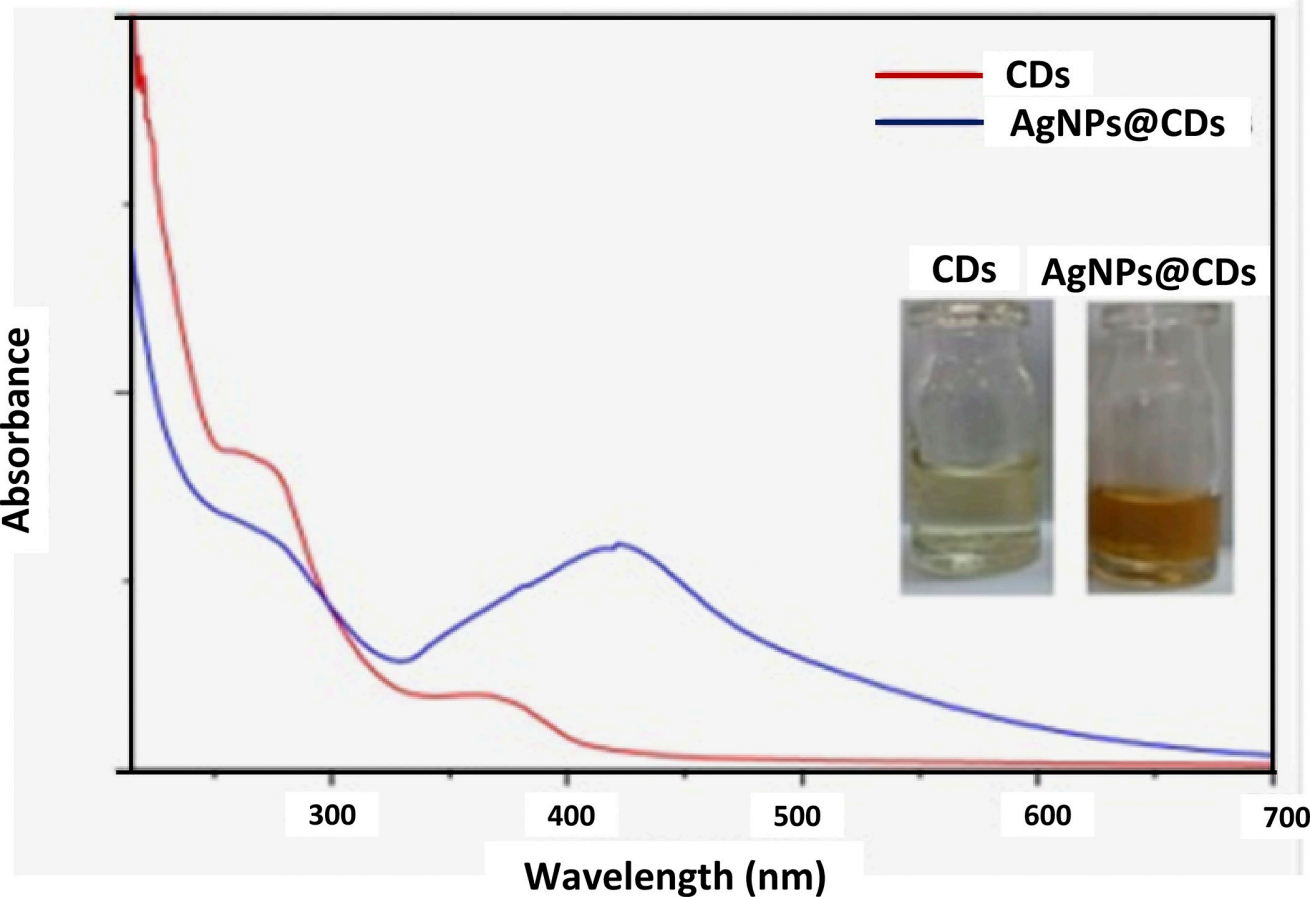
UV-Vis absorption spectra of Carbon nanodots and CD-AgNPs.

UV-Vis absorption spectra of manufactured Carbon nanodots revealed a prominent local superficial plasma resonance (LSPR) peak at 205 nm (**Fig 5**). Absorbance spectrum of CD-AgNPs revealed two prominent peaks at 255 nm and 205 nm that were ascribed to carbon nanodots whereas the LSPR absorption of the spherical form of silver nanoparticles was revealed by a broad peak at 408 nm.

### Fourier transformation infra-red (FTIR) spectroscopy

**Fig 6:**
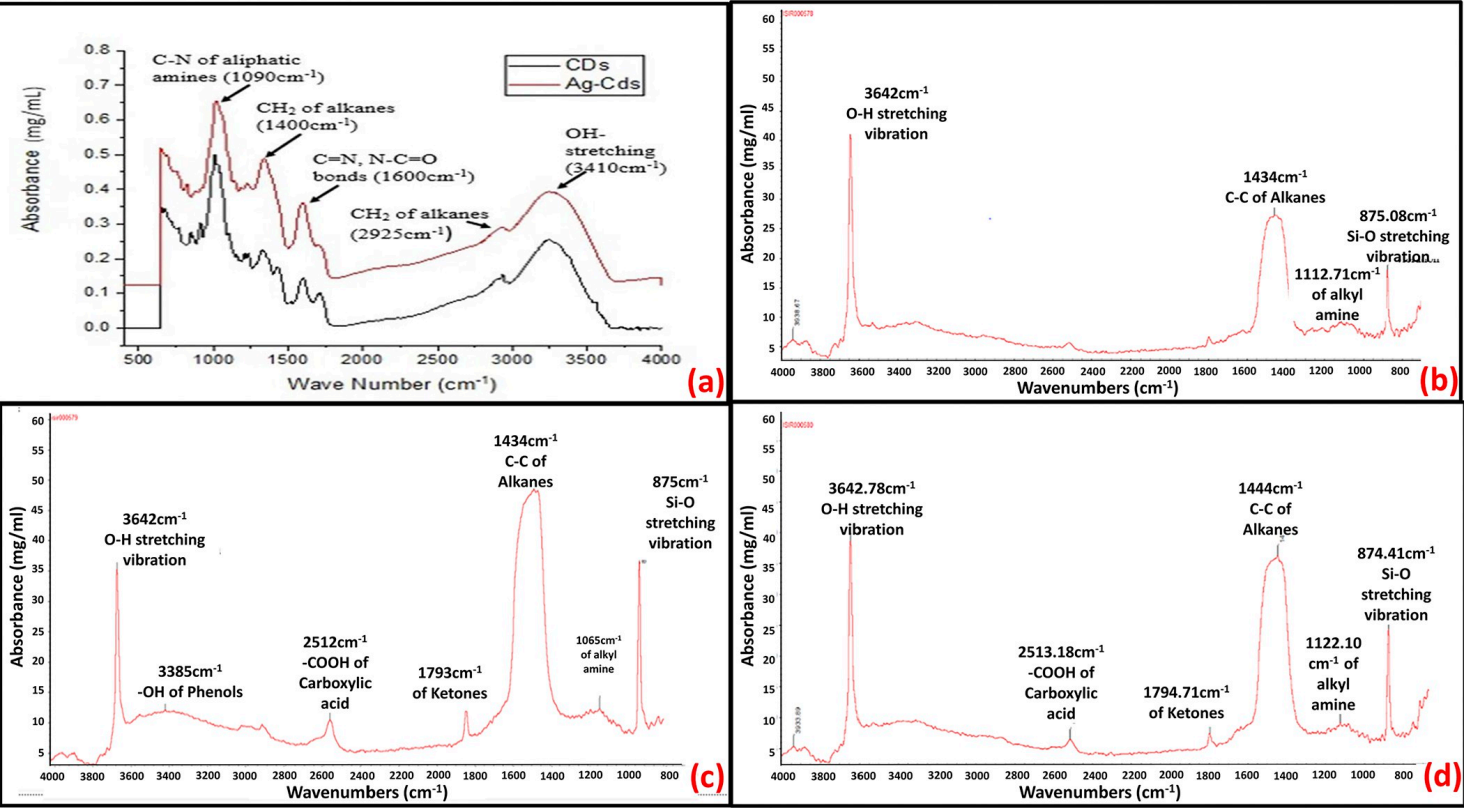
FTIR spectra showing peaks corresponding to different functional groups; (a) Carbon nanodots (CDs) and CD-AgNPs; (b) Control group; (c) Experimental endodontic sealer, E1; (d) Experimental endodontic sealer, E2.

FTIR spectra of Carbon nanodots as shown in **Fig 6(A)**, show an absorption band at 3410 cm^-1^ which matches the stretching vibration of -OH or N-H groups. Peaks at 2925 cm^-1^ represent CH_2_ of alkanes, the peaks at 1600 cm^-1^ represent the C = N or N-C = O bond of amide, and the peaks at 1400 cm^-1^ represent CH_2_ of alkanes or symmetric stretching of–COOH carboxyl group, the peaks at 1090 cm^-1^ represents C-N of aliphatic amines or C-O-C stretching and C-OH bending modes of the glycosidic bonds. The absorptions band between 800–430 cm^-1^ indicates the presence of C-H bending patterns for alkenes and aromatic structures, phenolic compounds, or N-H of deformation amines. FTIR spectra of the control group as shown in **Fig 6(B**) depict that at 3642 cm^-1^, O-H stretching vibrations are present. At 1434 cm^-1^, the C-C of alkanes is observed. At 1112.71 cm^-1^, alkyl amines are observed whereas the absorption peak at 875.08 cm^-1^ shows Si-O stretching vibration. The FTIR spectrum of E-1 as shown in **Fig 6(C)** depicts O-H stretching vibration at 3642 cm^-1^. At 3385 cm^-1^, -OH of phenols is observed. Absorption peak at 2512 cm^-1^ shows–COOH of carboxyl acid, at 1793 cm^-1^, R_2_C = O of ketones are observed. Absorption peaks at 1434 cm^-1^, 1065 cm^-1,^ and 875 cm^-1^ show C-C of alkanes, alkyl amines, and Si-O stretching vibrations respectively. The FTIR spectrum of E-2 as shown in **Fig 6(D**) depicts O-H stretching vibration at 3642.78 cm^-1^. The absorption peak at 2513.18 cm^-1^ shows–COOH of carboxyl acid, at 1794.71 cm^-1^, R_2_C = O of ketones are observed. Absorption peak at 2512 cm^-1^ shows the COOH group of carboxyl acid, at 1793 cm^-1^, R_2_C = O of ketones are observed. The absorption peaks at 1434 cm^-1^, 1065 cm^-1,^ and 875 cm^-1^ show C-C of alkanes, alkyl amines, and Si-O stretching vibrations respectively.

### Anti-microbial analysis

**Fig 7:**
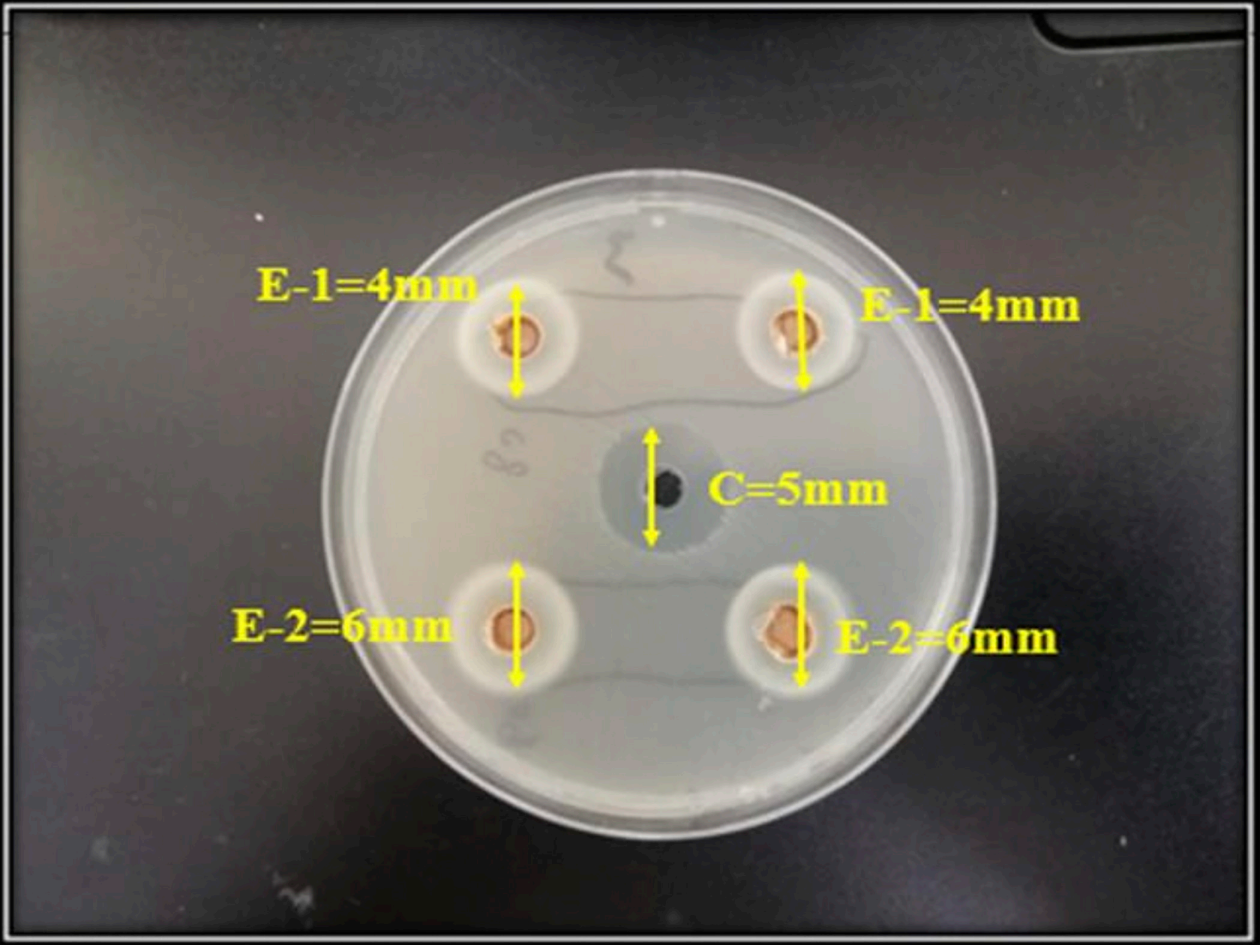
Zones of inhibition of groups C, E1, and E2 against *E*. *faecalis*.

**Fig 8:**
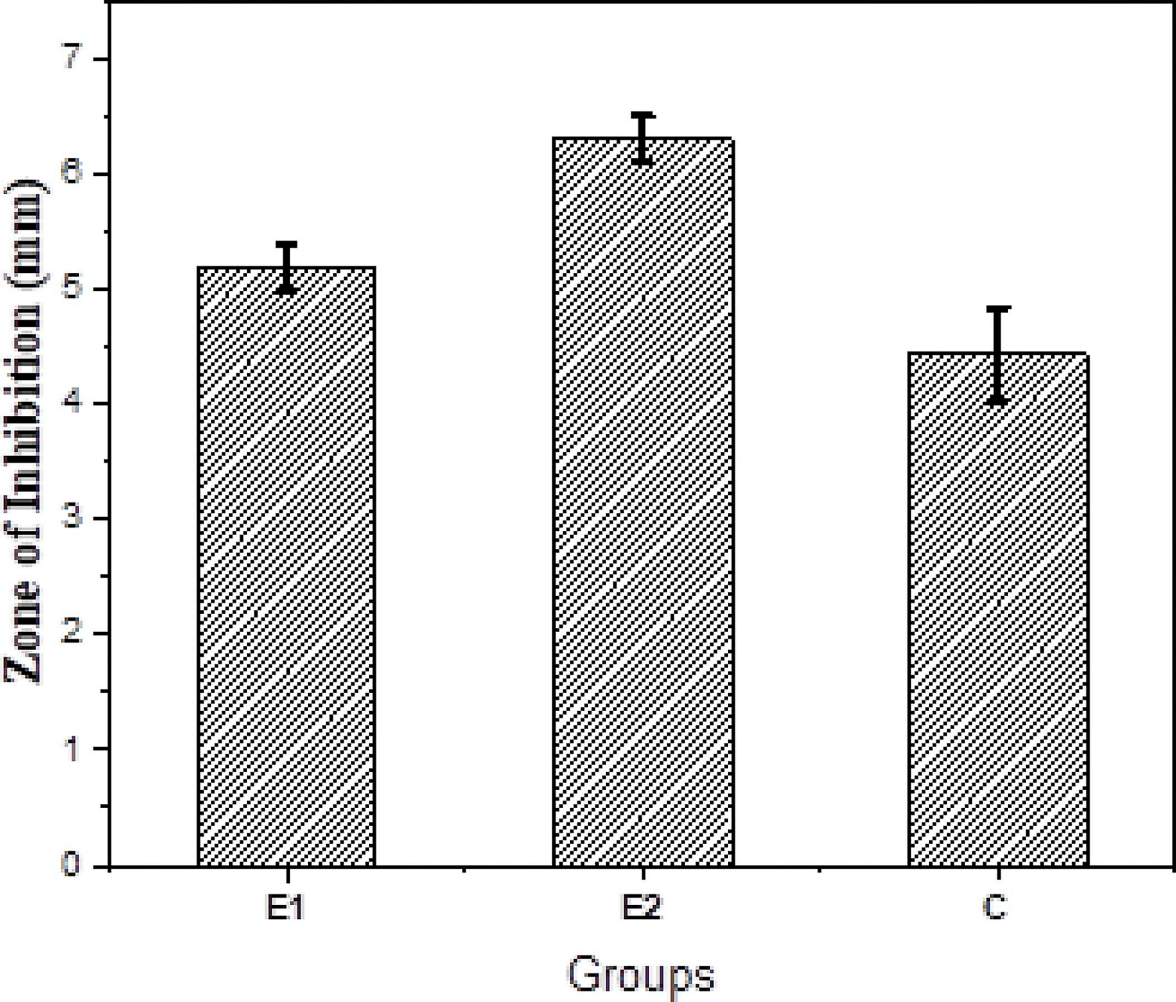
Values of the zone of inhibition (mean and standard deviation) against *E*. *faecalis*.

ZOIs of all the experimental endodontic sealers are shown in **Figs 7 and 8**. The experimental group, E-2 showed the greatest ZOI against *E*. *faecalis* (6.3±2 mm) whereas the control group, C showed the least ZOI against E. faecalis (4.43±2 mm). Statistically, a significant difference in the anti-bacterial activity against *E-faecalis* was observed within and between all tested groups *(p value = 0*.*001)*.

### Release kinetics of endodontic sealers

**Fig 9:**
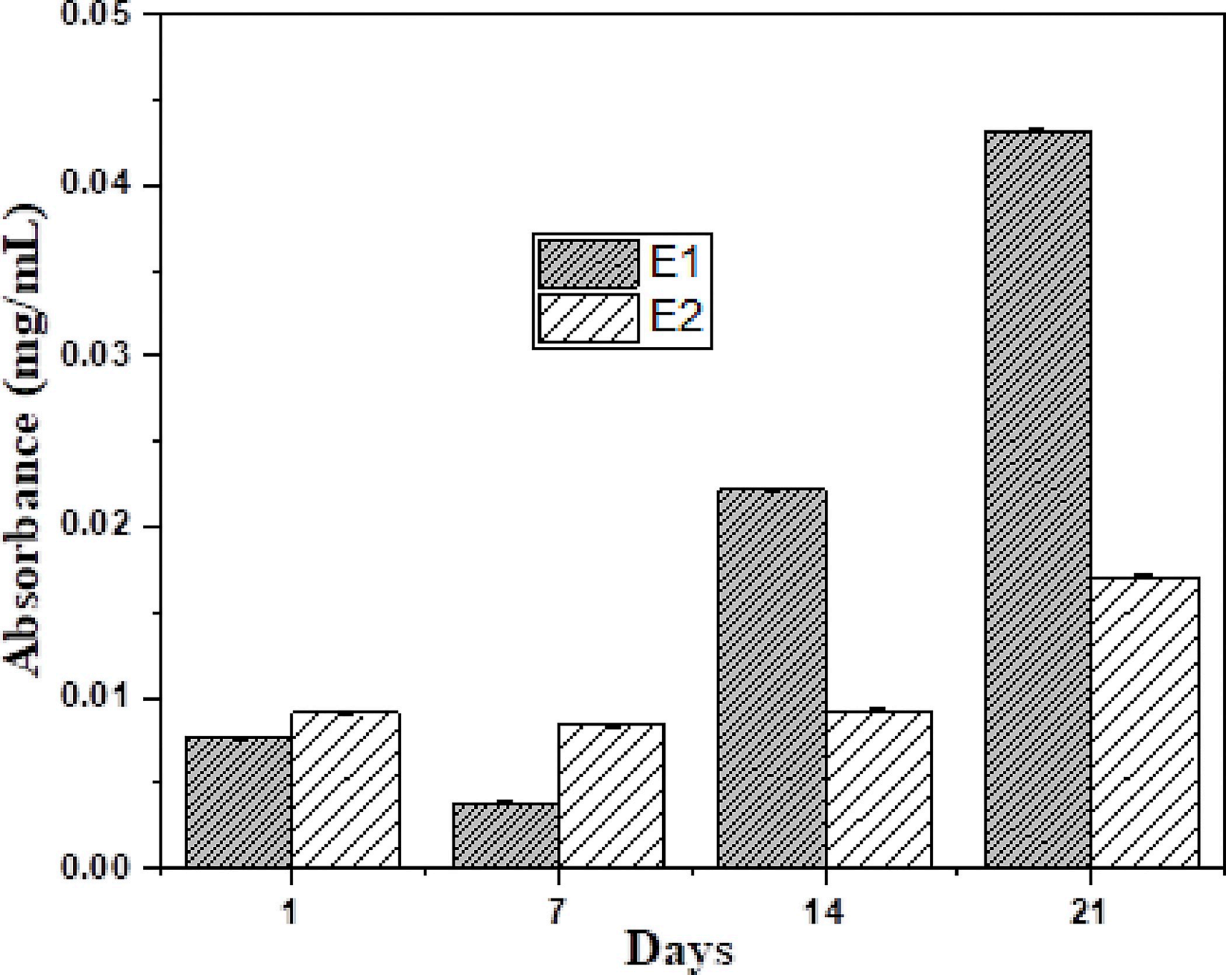
Release of silver ions at different time intervals from experimental groups i.e., E1 and E2.

The control group was devoid of CD-AgNPs, therefore, it did not exhibit any elusion of Ag+ ions. Within the experimental groups, the highest elusion of Ag+ ions were exhibited by E-1 (0.043±0.0001 mg/ml) and the lowest elusion of silver ions was exhibited by E-2 (0.017±0.001 mg/ml). Silver ions released at planned time intervals for 21 days for experimental groups are displayed in Fig 9. A statistically significant difference in release kinetics of silver ions (mg/ml) was observed within and between the control group (C) and all the experimental groups, i.e. E-1 and E-2 at all the planned time intervals for 21days *(p value = 0*.*000)*.

### Water solubility

**Fig 10:**
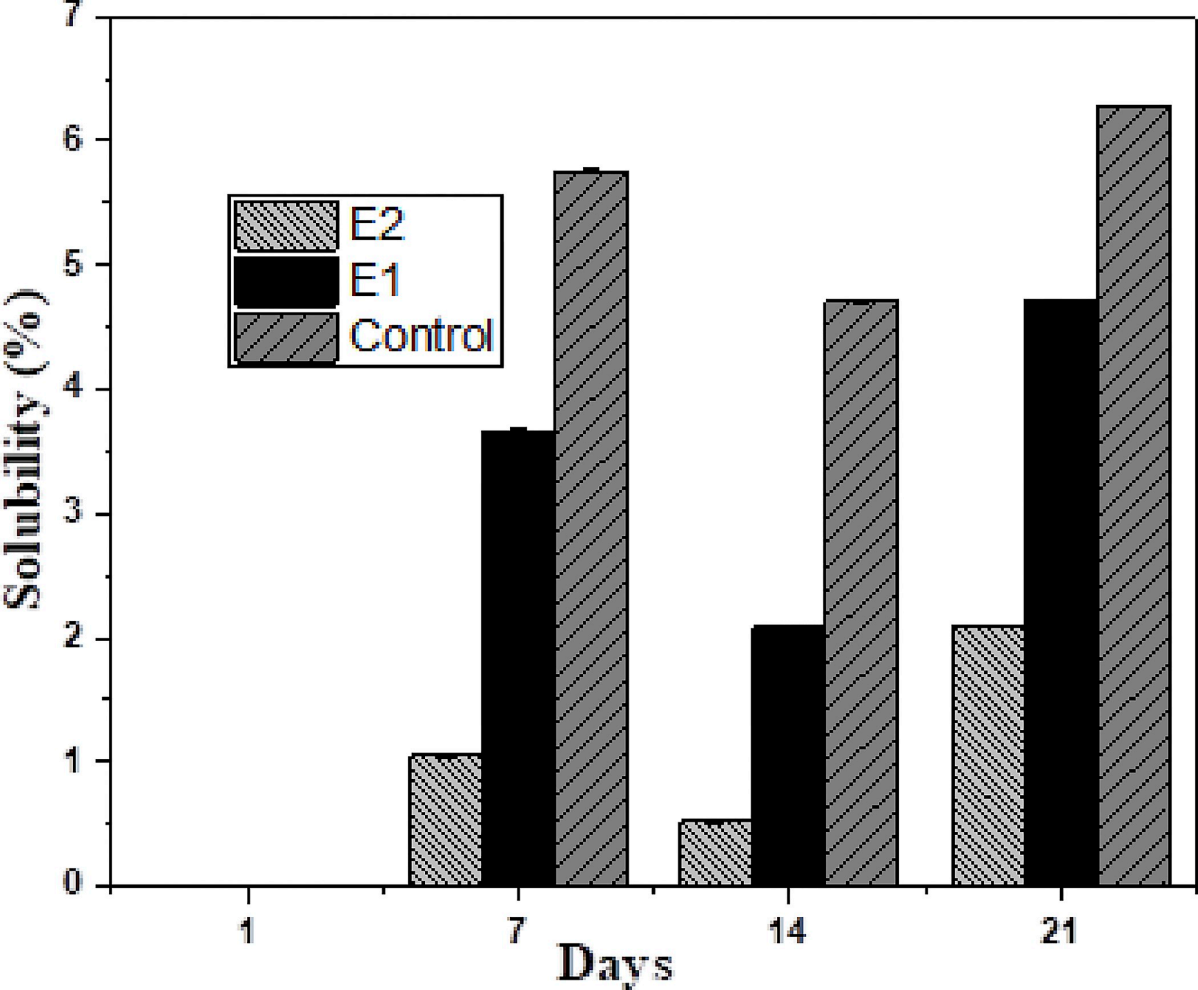
Water solubility of endodontic sealers sample discs at different time intervals.

The control group showed maximum percentage weight loss at all the time intervals and E-2 showed minimal weight loss at all the time intervals (Fig 10). A statistically significant difference in water solubility of the prepared sample discs was observed within and between all groups of this study at all the time intervals *(p value = 0*.*000)*.

## Discussion

Calcium hydroxide is one of the frequently employed endodontic medicaments due to its strong bactericidal activity. Moreover, a calcium hydroxide root canal sealer can promote calcification and repair of the dentinal tissue [22]. Calcium hydroxide sealer also promotes osteogenesis and cementogenesis at the apical barrier. This dual function of calcium hydroxide root canal sealer makes it quite useful in endodontic therapy. It not only enables the development of a tight seal between the core obturation material and the root canal space but also provides repair of the periapical tissue [4]. Calcium hydroxide endodontic sealer has been used in combination with different antibiotics [23]. *Enterococcus faecalis* is the species that is the most frequent cause of failed endodontic treatment [24]. *E*. *faecalis* in endodontic treated teeth is a frequent cause of recurrent and persistent endodontic infection as this bacteria is able to evade the antibacterial action of calcium hydroxide [5].

Antibiotic resistance in microbes advocated for a need to develop novel antibiotic-free antimicrobial products [8]. Nanoparticles are novel antimicrobial agents which can be incorporated into endodontic sealers to combat drug-resistant microbes [9]. Various kinds of nanoparticles i.e., include zinc oxide, copper oxide, chitosan, and silver nanoparticles, are available which can be incorporated into calcium hydroxide endodontic sealer [25]. The choice of AgNPs to be used in combination with the calcium hydroxide endodontic sealer was made because AgNPs demonstrate excellent antibacterial activity against *E*. *faecalis* [5, 26]. AgNPs stand out amongst antibacterial nanoparticles because of their ability to mediate a loss of chemical equilibrium in the bacterial cell [27]. AgNPs have been reported to generate ROS, leading to the degradation of membrane proteins and lipids [8], so, there is a need to stabilize AgNPs [28]. The solution to the generation of ROS by AgNPs is substitution by carbon nanodots which are bioactive and have they the ability to scavenge ROS and thus, stabilize AgNPs [28]. This is done through the generation of -COOH and–OH groups that exist on the surface of carbon nanodots. These groups stabilize the AgNPs by forming bonds with them [12].

In this study, the synthesis of the CD-AgNPs was done through a specific technique recommended by Gul et al., [13]. Carbonized banana peels were used as a source of carbon nonadots whereas AgNO_3_ was used as a source for silver. The MIC value of CD-AgNPs stock solution was found to be 5 mg/ml by employing the Broth Microdilution procedure in line with the Clinical and Laboratory Standard Institute (CLSI) guidelines. These findings are comparable to the results of Krishnan et al. who demonstrated the MIC of AgNPs against *E*. *faecalis* to be 5 mg/ml using serial broth microdilution method following CLSI guidelines [5]. Considering this MIC value (5 mg/ml), CD-AgNPs in two dissimilar concentrations, i.e. (5 mg/ml and 10 mg/ml) were used as liquid component to formulate novel calcium hydroxide based endodontic sealants. The calcium hydroxide powder and liquid components were mixed in a ratio of 1:1 as suggested by Almaimouni et al., 2020 [29] to produce disc samples (7 mm diameter and 3mm height).

SEM analysis of CD-AgNPs revealed spherical shaped nanoparticles with dimensions of 15–20 nm. The nanoparticles were uniformly distributed and also showed aggregation [30]. Various studies have reported the size and shape of AgNPs. Moreover, Neupane et al. reported AgNPs to be of spherical shape with size less than 50 nm [31]. Tailor et al. reported the size of AgNPs as 15.6 nm. Spherical shaped particles with aggregation were found [32]. Skiba et al. determined the spherical shape of AgNPs with sizes up to 15 nm. Moreover, aggregation of nanoparticles was also observed [33]. All these studies are following the findings of this study regarding the shape, size, and aggregation of AgNPs clusters. The EDS analysis of the CD-AgNPs stock solution employed in this study revealed that all the elements which were shown in the results are constituents of the different reagents used to synthesize the nanoparticle solution and the synthesized material did not contain any impurities in its chemical structure.

UV-Vis Spectroscopy of the carbon nanodots and CD-AgNPs was carried out in this study. Along with the larger shoulder band, the UV spectra of manufactured Carbon nanodots revealed a prominent localized surface plasma resonance (LSPR) peak at 205 nm which suggests that Carbon nanodots come in a wider range of sizes. These peaks were attributed to the transition of bonds, i.e. n- π* transitions of aromatic C = O bonds as well as π-π* transitions of aromatic C = C bonds. The absorbance spectrum of CD-AgNPs revealed two peaks at 255 nm and 205 nm that were attributed to Carbon nanodots. LSPR absorption of the spherical form of AgNPs was revealed by a broad peak at 408 nm. The samples were of a brown-reddish color, thus, indicating the presence of AgNPs. The optical properties of Carbon nanodots have been previously evaluated through UV-Vis Spectroscopy and found similar results as reported in this study [34, 35]. The Carbon nanodots synthesized by Athinarayanan et al. showed an optical absorption peak at 285 nm. It also evaluated the π–π* transition of aromatic units or nonbonding electrons [34]. In another study done by Kim et al., the UV–vis absorption spectrum of the carbon nanodots showed two sharp absorption peaks at 290 nm and 294 nm which were because of π-π* transition of C = C bonds [35]. UV-Vis absorption spectra of AgNPs in a study by Kahrilas et al. showed absorption peaks occurring between 380–420 nm. These were due to the size of AgNPs generated as well as the LSPR of the AgNPs. AgNPS have an inherent property of plasmon resonance which arises due to the coupling between incident electromagnetic radiation and electron cloud on AgNPs surface [36]. This is following this study’s findings. AgNPs synthesized by Kahrilas et al., in 2014 showed broad absorbance at λ_max_ = 425nm for AgNPs synthesized from tangelo and orange rind extract and λ_max_ = 403nm for AgNPs synthesized from grapefruit rind extract, which indicated successful biosynthesis of AgNPs. The samples exhibited deep reddish-amber shade, thus, demonstrating the existence of AgNPs [36]. All these findings are following this study’s findings.

Fourier Transform Infrared Spectra of Carbon nanodots and CD-AgNPs provide sufficient information about chemical structure of chemicals utilized in this study. The absorbance peaks of the FTIR spectrum indicate the participation of amino, carboxyl, hydroxyl, and carbonyl groups in the synthesis of these nanoparticles. These findings are similar to the ones stated by Gul et al. who found similar absorption peaks [13]. Rosman et al. performed FTIR of AgNPs and found that the absorption bands at 3381.17 cm^−1^ were due to the stretching of the phenols and O–H group of carboxylic acids [37].

The SEM images of the experimental endodontic sealer discs reveal irregular aggregated nanoparticles with a porous and uneven structure. The hexagonal shape of nanoparticles was observed. Channels and pores were also observed in the structure. These characteristic irregular, porous, hexagonal crystals of calcium hydroxide nanoparticles and aggregated structure have been revealed in prior studies by Chen et al., and Weththimuni et al. [38, 39]. The sample surface was rough/uneven [39] similar to our findings.

The EDS analysis of all the experimental groups synthesized in this study revealed that all the elements which were shown in the results are constituents of the different reagents used in this study and the synthesized materials did not contain any impurities in their chemical structure. The experimental groups were composed of Carbon, Calcium, Silver, and Oxygen. It was determined that in group E-1, the concentration of Carbon and Silver was lower as compared to group E-2. The concentration of Calcium in both the experimental groups remained the same.

Fourier Transform Infrared Spectra of the endodontic sealers used in this study give all the necessary information about chemical structure of the chemical reagents used in this study. The narrow absorption band at 3642 cm^-1^ is due to covalent bonds and stretching vibrations of the O-H group as reported in the literature [40]. In this study, for group E-1, at 3385 cm^-1^, -OH of phenols is observed. The absorption peak at 2512 cm^-1^ shows–COOH of carboxyl acid, at 1793 cm^-1^, R_2_C = O of ketones are observed. For group E-2, the absorption peak at 2513.18 cm^-1^ shows–COOH of carboxyl acid, at 1794.71 cm^-1^, R_2_C = O of ketones are observed. Absorption peak at 2512 cm^-1^ shows the COOH group of carboxyl acid. FTIR spectra of the control group do not contain the aforementioned peaks. This shows that E-1 and E-2 exhibit the specific absorption peaks due to the presence of CD-AgNPs in them whereas the control group did not contain CD-AgNPs, So, it did not exhibit those specific peaks.

In this study, agar well diffusion assay provided prominent, well-defined zones of inhibition against *E*. *faecalis* for all experimental groups. These findings are similar to those described by Heyder, 2013 and Elsaka, 2012 [41, 42]. Heyder al. reported that *E*. *faecalis* is not suppressed by calcium hydroxide alone [41] which is following this study for the control group but calcium hydroxide reported significant antibacterial activity against *E*. *faecalis* when it was used in association with some antibacterial material [42] which is true for experimental groups E1 and E2. In this study, the antibacterial effectiveness of CD-AgNPs was demonstrated against *E*. *faecalis*. These findings are similar to those described in the literature by Wong et al. where the increased antimicrobial activity of calcium hydroxide when blended with AgNPs was reported than calcium hydroxide alone [10]. Moreover, studies have reported that Calcium hydroxide mixed with AgNPs has strong antibacterial activity against *E*. *faecalis* [43, 44]. Tülü et al. reported that the effectiveness of calcium hydroxide used in combination with AgNPs was far superior to calcium hydroxide employed alone [43]. Vilelea Teixeira et al., in their study stated that adding AgNPs to root-canal sealers greatly enhanced the antibacterial action against *E*. *faecalis* [44]. Henceforth, present study confirms the fact that the antibacterial action of calcium hydroxide based endodontic sealers containing CD-AgNPs was significantly enhanced as compared to calcium hydroxide alone, thus, highlighting the excellent, antibacterial potential of AgNPs as stated in literature.

Outcomes of this work suggested that the release rate of Ag+ ions from all experimental groups was less than 0.01 mg/ml at all the time intervals. E-1 exhibited an early explosive elution of Ag^+^ ions and then a steady release of Ag^+^ ions throughout 21 days whereas E-2 exhibited a steady elution of Ag^+^ ions throughout the period of 21 days. This steady release is of utmost clinical significance because it shows that the main antibacterial agent, Ag^+^ ions are available steadily throughout the tested experimental period of 21 days. Hence, it is stated that a steady antibacterial activity will occur against *E*. *faecalis*, the main causative agent responsible for recurrent and persistent root canal infection. At all the time intervals, the release rate of AgNPs was less than 0.01 mg/ml. This is following the European Union Legislation which postulates that the release rate of AgNPs should be less than the permitted amount (0.01 mg/ml) [45, 46]. In this study, a steady release of AgNPs from the calcium hydroxide sealer structure was observed. These outcomes are comparable to those stated by Ballesteros et al. who evaluated the release of AgNPs from the AgNPs-nanogel structure and found the controlled and steady release of AgNPs over 21 days [27]. In the present study, the experimental groups did not show significant dissolution when immersed in water for 21 days in comparison to the control group. Highest value of solubility was exhibited by control group (6.21%) and the lowest water solubility was exhibited by E-2 (4.71%). This is following the fact that calcium hydroxide sealer can endure long term exposure to tissue fluids without undergoing remarkable dissolution [47]. The outcomes of this work advocate that by the integration of CD-AgNPs in calcium hydroxide-based sealers, the solubility of the experimental novel endodontic sealers was lowered over 21 days. This ascertains the fact that CD-AgNPs significantly lowered the solubility of the novel root-canal sealers. Findings of this study also determine <3% solubility at 24 hours. This result is following the protocol of ISO 6876:2012 regarding the solubility of endodontic sealers; (<3% solubility at 24 hours is the standard). In this study, the control and experimental groups exhibited 0% solubility at 24 hours, thus, meeting the ISO standard. These findings are comparable to a study done by Silva et al. which reported a percentage weight loss of calcium hydroxide sealer of 0.29% after 24 hours [48]. Zordan-Bronzel et al. reported somewhat different results of water solubility of calcium hydroxide sealers. On day 7 and day 28, the solubility of tested endodontic sealers was reported to be 7.48% and 10.84% respectively [49]. In another study done by Poggio et al., it was reported that calcium hydroxide sealers exhibited water solubility of less than 3% at 24 hours. It was also reported that calcium hydroxide sealers are soluble over time, thus, emphasizing the need to strengthen them by the addition of another material [50].

According to the findings of our study, the novel endodontic sealer exhibited adequate antibacterial activity against *E*. *faecalis*. This suggests that by using this material in endodontic treatment, *E*. *faecalis* can be effectively eradicated from the root canals; thus, growing the rate of success of root canal treatments. Moreover, a steady release of silver ions has been found with a solubility of <3% after 24 hours. This implies that a steady antibacterial effect will be observed in future clinical applications. Henceforth, after performing in-vitro studies for the assessment of mechanical and optical properties of this material on the tooth structure, this novel synthesized material can be an adequate candidate for clinical use in the future. It has the potential to bring about revolutionary changes in combating the primary causative agent of recurrent and persistent endodontic infection- *E*. *faecalis*. Further tests are required to analyze this material for clinical use in the future.

The enhanced antibacterial activity shown by the novel endodontic sealer makes it suitable for use in future clinical applications in endodontic treatment. Since this synthesized material exhibited a steady release of silver ions and water solubility of less than 3% after 24 hours, it can be postulated that a steady supply of this antibacterial agent will be available in the root canals and will be useful in the complete eradication of *E*. *faecalis* from the root canals declining the cases of endodontic failure.

### Conclusions

Within the scope of this study, it can conclude that CD-AgNPs were effectively synthesized, exhibiting a MIC of 5 mg/ml against *E*. *faecalis* and cytocompatibility of 84.57% with *NIH3T3* cell lines. The novel endodontic sealers displayed a consistent release of silver ions, a water solubility of less than 3% after 24 hours following the ISO standard, and enhanced antibacterial potential against *E*. *faecalis*.

## Supporting information

S1 File Statistical data of the manuscript. (PDF)
