# Supplementary material for: Biochemical properties of novel Carbon nanodot-stabilized silver nanoparticles enriched calcium hydroxide endodontic sealer
Source: PLoS One. 2024 Jul 3;19(7):e0303808. doi: 10.1371/journal.pone.0303808 (PMC11221646; doi:10.1371/journal.pone.0303808)
Supplement: S1 File — (PDF) [file pone.0303808.s001.pdf]

Data of the Article

APPENDIX A

PILOT STUDY

A pilot study was done in which CD-stabilized AgNPs were successfully synthesized by microwave irradiation process (the methodology and results of which are given on page no. 41 and 56 respectively) and FTIR was done to characterize them (the methodology and results of which are given on page no. 44 and 62 respectively). Then, MIC determination of CD-stabilized AgNPs was done against *E. faecalis* (the methodology and results of which are given on page no. 45 and 66 respectively).

After finding out the MIC value, CD-stabilized AgNPs were taken in two different concentrations of 5mg/mL and 10mg/mL which were used as liquid portion to be mixed in pure calcium hydroxide powder to produce the experimental groups E1 and E2 respectively. Next, determination of the optimum ratio of calcium hydroxide powder and CD-stabilized AgNPs solution was carried out to obtain the workable consistency for endodontic sealers. For this Calcium hydroxide powder was weighed on an electronic balance and then mixed with CD-stabilized AgNPs solution in different experimental ratios. It was determined that the optimum liquid to powder ratio was 1:1.5 because at this particular ratio, the endodontic sealer completely set to form a smooth disc after pouring in a Teflon mould. At a liquid to powder ratio of 1:1, the sealer did not set to form a smooth disc. Therefore, 1:1.5 was selected as the optimum liquid to powder ratio.

After finalizing the optimum liquid powder ratio, the samples discs (7mm diameter and 3mm height) were prepared. After thorough mixing, the resultant paste was then poured into a Teflon mould of dimensions (7mm diameter and 3mm height). These were then placed in a desiccator overnight to ensure complete drying of the sample discs. After this, the sample discs were removed from the Teflon mould and they were trimmed to adjust the dimensions. Next, careful labelling of the sample discs was done followed by weighing on an electronic balance.

APPENDIX B

ZONE OF INHIBITION

Descriptives

zone_of_inhibition

	N	Mean	Std. Deviation	Std. Error	95% Confidence Interval for Mean Lower Bound	95% Confidence Interval for Mean Upper Bound	Minimum	Maximum
Control	3	4.4333	.40415	.23333	3.4294	5.4373	4.00	4.80
E1	3	5.2000	.20000	.11547	4.7032	5.6968	5.00	5.40
E2	3	6.3000	.20000	.11547	5.8032	6.7968	6.10	6.50
Total	9	5.3111	.84918	.28306	4.6584	5.9639	4.00	6.50

ANOVA

zone_of_inhibition

	Sum of Squares	df	Mean Square	F	Sig.
Between Groups	5.282	2	2.641	32.562	.001
Within Groups	.487	6	.081		
Total	5.769	8			

Multiple Comparisons

Dependent Variable: zone_of_inhibition

Tukey HSD

(I) Group	(J) Group	Mean Difference (I-J)	Std. Error	Sig.	95% Confidence Interval Lower Bound	95% Confidence Interval Upper Bound
Control	E1	-.76667*	.23254	.038	-1.4802	-.0532
	E2	-1.86667*	.23254	.000	-2.5802	-1.1532
E1	Control	.76667*	.23254	.038	.0532	1.4802
	E2	-1.10000*	.23254	.008	-1.8135	-.3865
E2	Control	1.86667*	.23254	.000	1.1532	2.5802
	E1	1.10000*	.23254	.008	.3865	1.8135

*. The mean difference is significant at the 0.05 level.

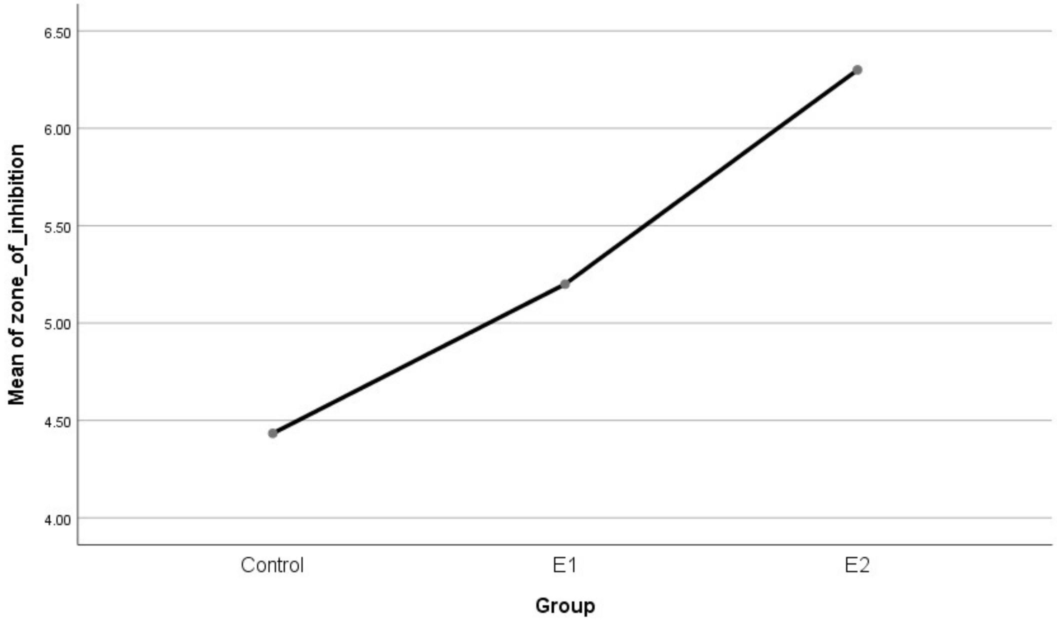

WATER SOLUBILITY

Descriptive Statistics

	Group	Mean	Std. Deviation	N
Water_Solubility_at_day_1	Control	.0000	.00000	3
	E-1	.0000	.00000	3
	E-2	.0000	.00000	3
	Total	.0000	.00000	9
Water_Solubility_at_day_7	Control	2.0900	.01000	3
	E-1	1.0500	.01000	3
	E-2	.5200	.01000	3
	Total	1.2200	.69174	9
Water_Solubility_at_day_14	Control	4.7100	.01000	3
	E-1	3.6600	.01000	3
	E-2	2.0900	.01000	3
	Total	3.4867	1.14195	9
Water_Solubility_at_day_21	Control	6.2800	.01000	3
	E-1	5.7633	.00577	3
	E-2	4.7100	.01000	3
	Total	5.5844	.69298	9

Tests of Within-Subjects Effects

Measure: Solubility

Source		Type III Sum of Squares	Df	Mean Square	F	Sig.
Time	Sphericity Assumed	165.191	3	55.064	683547.724	.000
	Greenhouse-Geisser	165.191	1.524	108.359	683547.724	.000
	Huynh-Feldt	165.191	2.619	63.080	683547.724	.000
	Lower-bound	165.191	1.000	165.191	683547.724	.000
Time * Group	Sphericity Assumed	5.622	6	.937	11630.897	.000
	Greenhouse-Geisser	5.622	3.049	1.844	11630.897	.000
	Huynh-Feldt	5.622	5.237	1.073	11630.897	.000
	Lower-bound	5.622	2.000	2.811	11630.897	.000
Error(Time)	Sphericity Assumed	.001	18	8.056E-5		
	Greenhouse-Geisser	.001	9.147	.000		
	Huynh-Feldt	.001	15.712	9.228E-5		
	Lower-bound	.001	6.000	.000		

Tests of Between-Subjects Effects

Measure: Solubility

Transformed Variable: Average

Source	Type III Sum of Squares	df	Mean Square	F	Sig.
Intercept	238.291	1	238.291	6598818.769	.000
Group	12.479	2	6.239	172785.538	.000
Error	.000	6	3.611E-5		

Pairwise Comparisons

Measure: Solubility

(I) Group	(J) Group	Mean Difference (I-J)	Std. Error	Sig.[b]	95% Confidence Interval for Difference[b]	
					Lower Bound	Upper Bound
Control	E-1	.652*	.002	.000	.644	.660
	E-2	1.440*	.002	.000	1.432	1.448
E-1	Control	-.652*	.002	.000	-.660	-.644
	E-2	.788*	.002	.000	.780	.796
E-2	Control	-1.440*	.002	.000	-1.448	-1.432
	E-1	-.788*	.002	.000	-.796	-.780

Based on estimated marginal means

*. The mean difference is significant at the .05 level.

a) Adjustment for multiple comparisons: Bonferroni.

Pairwise Comparisons

Measure: Solubility

(I) Time	(J) Time	Mean Difference (I-J)	Std. Error	Sig.[b]	95% Confidence Interval for Difference[b]	
					Lower Bound	Upper Bound
1	2	-1.220*	.003	.000	-1.233	-1.207
	3	-3.487*	.003	.000	-3.500	-3.474
	4	-5.584*	.003	.000	-5.596	-5.573
2	1	1.220*	.003	.000	1.207	1.233
	3	-2.267*	.006	.000	-2.291	-2.242
	4	-4.364*	.005	.000	-4.383	-4.346
3	1	3.487*	.003	.000	3.474	3.500
	2	2.267*	.006	.000	2.242	2.291
	4	-2.098*	.004	.000	-2.111	-2.084
4	1	5.584*	.003	.000	5.573	5.596
	2	4.364*	.005	.000	4.346	4.383

3	2.098*	.004	.000	2.084	2.111

Based on estimated marginal means

*. The mean difference is significant at the .05 level.

b. Adjustment for multiple comparisons: Bonferroni.

Multiple Comparisons

Measure: Solubility

Tukey HSD

(I) Group	(J) Group	Mean Difference (I-J)	Std. Error	Sig.	95% Confidence Interval Lower Bound	95% Confidence Interval Upper Bound
Control	E-1	.6517*	.00245	.000	.6441	.6592
	E-2	1.4400*	.00245	.000	1.4325	1.4475
E-1	Control	-.6517*	.00245	.000	-.6592	-.6441
	E-2	.7883*	.00245	.000	.7808	.7959
E-2	Control	-1.4400*	.00245	.000	-1.4475	-1.4325
	E-1	-.7883*	.00245	.000	-.7959	-.7808

Based on observed means.

The error term is Mean Square(Error) = 9.03E-006.

*. The mean difference is significant at the .05 level.

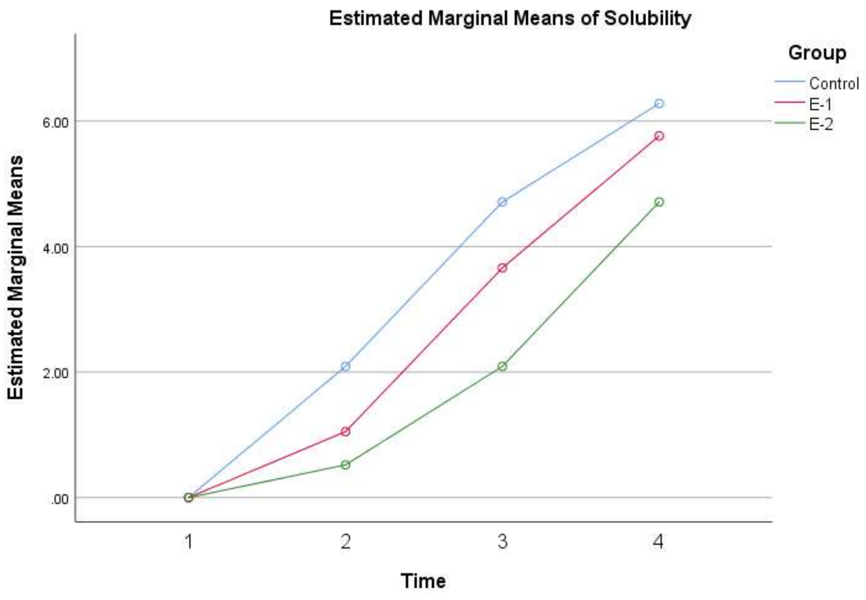

RELEASE KINETICS

Descriptive Statistics

	E_1, E_2, C	Mean	Std. Deviation	N
Release_day_1	E1	.007633	.0000577	3
	E2	.009100	.0001000	3
	C	.000000	.0000000	3
	Total	.005578	.0042317	9
Release_day_7	E1	.003800	.0001000	3
	E2	.008400	.0001000	3
	C	.000000	.0000000	3
	Total	.004067	.0036435	9
Release_day_14	E1	.022200	.0001000	3
	E2	.009200	.0001000	3
	C	.000000	.0000000	3
	Total	.010467	.0096600	9
Release_day_21	E1	.043200	.0001000	3
	E2	.017100	.0001000	3
	C	.000000	.0000000	3
	Total	.020100	.0188411	9

Tests of Within-Subjects Effects

Measure: Silver_Ions_Release

Source		Type III Sum of Squares	df	Mean Square	F	Sig.	Partial Eta Squared	Noncent. Parameter	Observed Power[a]
Time	Sphericity Assumed	.001	3	.000	121096.643	.000	1.000	363289.929	1.000
	Greenhouse-Geisser	.001	1.547	.001	121096.643	.000	1.000	187381.121	1.000
	Huynh-Feldt	.001	2.678	.001	121096.643	.000	1.000	324355.783	1.000
	Lower-bound	.001	1.000	.001	121096.643	.000	1.000	121096.643	1.000
Time * Groups	Sphericity Assumed	.002	6	.000	68891.929	.000	1.000	413351.571	1.000
	Greenhouse-Geisser	.002	3.095	.001	68891.929	.000	1.000	213202.389	1.000
	Huynh-Feldt	.002	5.357	.000	68891.929	.000	1.000	369052.270	1.000
	Lower-bound	.002	2.000	.001	68891.929	.000	1.000	137783.857	1.000
Error(Time)	Sphericity Assumed	7.000E-8	18	3.889E-9					
	Greenhouse-Geisser	7.000E-8	9.284	7.540E-9					
	Huynh-Feldt	7.000E-8	16.071	4.356E-9					
	Lower-bound	7.000E-8	6.000	1.167E-8					

a. Computed using alpha = .05

Tests of Between-Subjects Effects

Measure: Silver_Ions_Release
Transformed Variable: Average

Source	Type III Sum of Squares	df	Mean Square	F	Sig.	Partial Eta Squared	Noncent. Parameter	Observed Power[a]
Intercept	.004	1	.004	284720.891	.000	1.000	284720.891	1.000
Groups	.002	2	.001	87192.413	.000	1.000	174384.826	1.000
Error	7.667E-8	6	1.278E-8					

a. Computed using alpha = .05

Pairwise Comparisons

Measure: Silver_Ions_Release

(I) E_1, E_2, C	(J) E_1, E_2, C	Mean Difference (I-J)	Std. Error	Sig.[b]	95% Confidence Interval for Difference[b] Lower Bound	Upper Bound
E1	E2	.008*	.000	.000	.008	.008
	C	.019*	.000	.000	.019	.019
E2	E1	-.008*	.000	.000	-.008	-.008
	C	.011*	.000	.000	.011	.011
C	E1	-.019*	.000	.000	-.019	-.019
	E2	-.011*	.000	.000	-.011	-.011

Based on estimated marginal means

*. The mean difference is significant at the .05 level.

b. Adjustment for multiple comparisons: Bonferroni.

Pairwise Comparisons

Measure: Silver_Ions_Release

(I) Time	(J) Time	Mean Difference (I-J)	Std. Error	Sig.[b]	95% Confidence Interval for Difference[b] Lower Bound	Upper Bound
1	2	.002*	.000	.000	.001	.002
	3	-.005*	.000	.000	-.005	-.005
	4	-.015*	.000	.000	-.015	-.014
2	1	-.002*	.000	.000	-.002	-.001
	3	-.006*	.000	.000	-.007	-.006
	4	-.016	.000	.	-.016	-.016
3	1	.005*	.000	.000	.005	.005
	2	.006*	.000	.000	.006	.007
	4	-.010*	.000	.000	-.010	-.009
4	1	.015*	.000	.000	.014	.015
	2	.016	.000	.	.016	.016
	3	.010*	.000	.000	.009	.010

Based on estimated marginal means

*. The mean difference is significant at the .05 level.

b. Adjustment for multiple comparisons: Bonferroni.

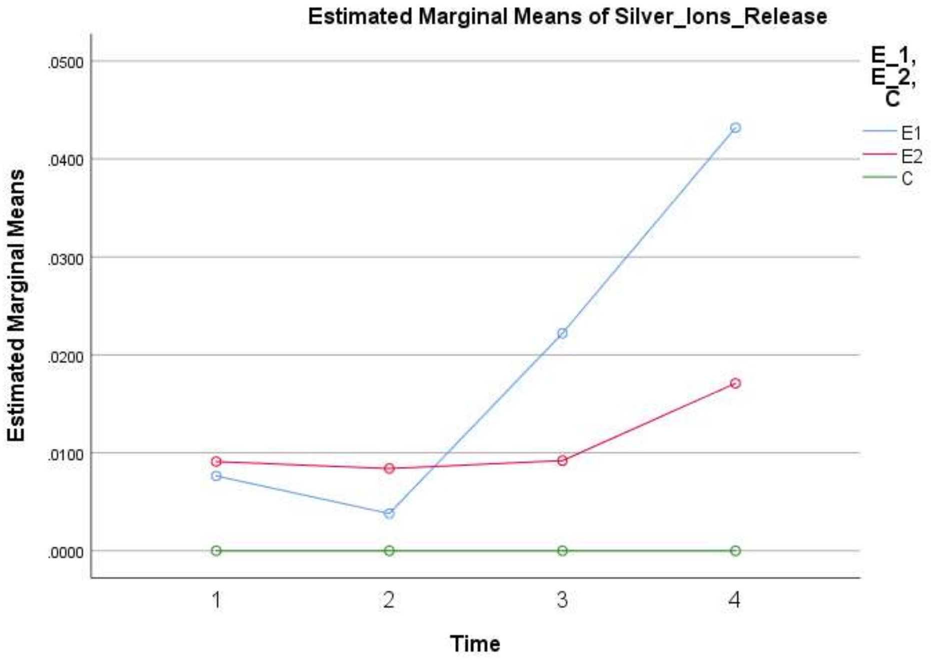

CYTOTOXICITY ANALYSIS

Descriptives

Cytotoxicity

	N	Mean	Std. Deviation	Std. Error	95% Confidence Interval for Mean Lower Bound	95% Confidence Interval for Mean Upper Bound	Minimum	Maximum
Control	3	99.9900	.01000	.00577	99.9652	100.0148	99.98	100.00
E1-5mg/mL	3	84.4700	.81872	.47269	82.4362	86.5038	83.61	85.24
E2-10mg/mL	3	82.8433	.43317	.25009	81.7673	83.9194	82.50	83.33
Total	9	89.1011	8.21006	2.73669	82.7903	95.4119	82.50	100.00

ANOVA

Cytotoxicity

	Sum of Squares	df	Mean Square	F	Sig.
Between Groups	537.525	2	268.762	939.692	.000
Within Groups	1.716	6	.286		
Total	539.241	8			

Multiple Comparisons

Dependent Variable: Cytotoxicity
Tukey HSD

(I) Groups	(J) Groups	Mean Difference (I-J)	Std. Error	Sig.	95% Confidence Interval Lower Bound	95% Confidence Interval Upper Bound
Control	E1-5mg/mL	15.52000*	.43666	.000	14.1802	16.8598
	E2-10mg/mL	17.14667*	.43666	.000	15.8069	18.4865
E1-5mg/mL	Control	-15.52000*	.43666	.000	-16.8598	-14.1802
	E2-10mg/mL	1.62667*	.43666	.023	.2869	2.9665
E2-10mg/mL	Control	-17.14667*	.43666	.000	-18.4865	-15.8069
	E1-5mg/mL	-1.62667*	.43666	.023	-2.9665	-.2869

*. The mean difference is significant at the 0.05 level.
